# Retraction: RASA1 inhibits the progression of renal cell carcinoma by decreasing the expression of miR-223-3p and promoting the expression of *FBXW7*

**DOI:** 10.1042/BSR-2019-4143_RET

**Published:** 2024-03-22

**Keywords:** FBWX7, miR-233-3p, RASA1, Renal cell carcinoma

This article is being retracted from *Bioscience Reports* at the request of the Editor-in-Chief and the Editorial Board following receipt of a notification from a reader, alerting the Editorial Office to multiple images within this paper that seem to appear in other papers by unrelated authors. Specifically:

- Figure 3D control panel which appears as Figure 4D sh-NC+VEGFA panel from Chen et al 2020 (DOI: 10.3389/fcell.2020.595585), and Figure 2D SPC-A1 NC panel from Zhang et al 2020 (DOI: 10.2147/CMAR.S268375) [retracted]
- Figure 5G ACHN NC+mimic NC and ACHN RASA1+mimic NC panels which appear as Figure 5E SPC-A1 NC and SPC-A1 sh-CASC9 panels from Zhang et al 2020 (DOI: 10.2147/CMAR.S268375) [retracted]
- Figure 5G 786-0 RASA1+miR-223-3p panel which appears as Figure 6I H1299 CDDP+miR-613 mimics+GJA1 panel from Luo et al 2021 (DOI: 10.3892/mmr.2021.12024) with hue alteration
- Figure 7F NC panel which appears as Figure 4F sh-Control panel from Chen et al 2020 (DOI: 10.2147/OTT.S253730) [retracted] with image rotation and hue alteration, and Figure 7D Ki-67 LINC01278 panel from Qu et al 2020 (DOI: 10.1002/jcp.29582) with image rotation
- Figure 7F si-RASA1 panel which appears as Figure 3F PCNA NC panel from Zhang et al 2020 (DOI: 10.2147/CMAR.S268375) [retracted] with image rotation, Figure 1D Chemosensitive tissues panel from Xu et al 2021 (DOI: 10.3892/mmr.2021.11902) with image rotation, Figure 4F sh-LINC01260 panel from Chen et al 2020 (DOI: 10.2147/OTT.S253730) [retracted] with image rotation and hue alteration, and Figure 7D PCNA LINC01278 panel from Qu et al 2020 (DOI: 10.1002/jcp.29582) with image rotation
- Figure 7F RASA1 panel which appears as Figure 1D Chemoresistant tissues panel from Xu et al 2021 (DOI: 10.3892/mmr.2021.11902 with image rotation, and Figure 7D PCNA vector panel from Qu et al 2020 (DOI: 10.1002/jcp.29582) with image rotation

The authors initially requested to retract the article stating they cannot locate or verify the original data for this paper, but now request to perform the experiments again to provide new data to address the concerns raised. However, due to concerns surrounding the scientific validity of this paper and duplications between the original data and other papers, the Editorial Board stands by the decision to retract this article.

